# The preemptive effects of oral pregabalin on perioperative pain management in lower limb orthopedic surgery: a systematic review and meta-analysis

**DOI:** 10.1186/s13018-022-03101-9

**Published:** 2022-04-13

**Authors:** Zhao Chen, Jialei Chen, Rong Luo, Jiabao Jiang, Zhou Xiang

**Affiliations:** Department of Orthopaedics, West China Hospital, Sichuan University, Guoxue Lane 37, Chengdu, 610041 Sichuan Province China

**Keywords:** Pregabalin, Perioperative pain management, Lower limb orthopedic surgery, Meta-analysis

## Abstract

**Background:**

To systematically review the literature and provide a comprehensive understanding of the preemptive effects of oral pregabalin on perioperative pain management in lower limb orthopedic surgery.

**Method:**

We searched three electronic databases for randomized controlled trials comparing the results of preoperative pregabalin and placebo in patients undergoing lower limb orthopedic surgery. Data analyses were conducted using RevMan 5.4.

**Results:**

Twenty-one randomized controlled trials met our inclusion criteria. The cumulative opioid consumption within 24 and 48 h postoperatively in the pregabalin group was significantly less than that in the placebo group. The pooled static pain intensity at all time points within the first day was significantly lower in the pregabalin group than in the placebo group. Lower dynamic pain intensity at 48 h was detected in the pregabalin group than in the placebo group. Meanwhile, pregabalin led to a lower incidence of nausea but appeared to be associated with a higher incidence of dizziness and sedation. Subgroup analyses showed that no difference was detected between subgroups stratified by dosing regimen or pregabalin dose in the results of opioid consumption, pain intensity and incidence of complications.

**Conclusion:**

This meta-analysis supports the use of pregabalin preoperatively in patients undergoing lower limb orthopedic surgery. However, it was wary of the resulting increase in dizziness and sedation. There is no evidence to support the continued use of pregabalin postoperatively or using more than 150 mg of pregabalin per day.

*Trial registration*: This study was registered on 09 November 2021 with INPLASY (registration number: INPLASY2021110031).

**Supplementary Information:**

The online version contains supplementary material available at 10.1186/s13018-022-03101-9.

## Background

Acute postoperative pain is a common problem faced by patients undergoing surgical treatment and arises after activation of nociceptors, inflammation, and nerve injury [1, 2]. Unrelieved postoperative pain may lead to prolonged hospital stay and recovery time [3, 4]. Moreover, approximately 10–50% of patients may develop chronic pain after surgery, which could further affect their quality of life [2]. Considering the increased opioid consumption and its adverse effects as well as the complications caused by poor postoperative pain control, multimodal analgesia has been recommended in pain management [5, 6].

Pregabalin can reduce the release of excitatory neurotransmitters and the excitability of synapses by inhibiting calcium influx through high-voltage gated channels [7, 8]. It has been proven that pregabalin can exert its analgesic effect by reducing the hyperexcitability of dorsal horn neurons caused by tissue damage rather than reducing pain transmission from the injury site [9, 10]. Considering its analgesic properties, preoperative administration of pregabalin has been widely used in perioperative pain management, and its analgesic effectiveness in procedures (such as spinal surgery) has been confirmed by existing meta-analyses [3, 11].

Pregabalin has been used in lower limb orthopedic surgery for more than 10 years, and several previous randomized trials have explored its effect on perioperative pain management [12–15]. However, there is no official consensus as to whether pregabalin is effective in perioperative pain management for patients undergoing lower limb orthopedic surgery. A meta-analysis is warranted.

In this analysis, we aim to conduct a meta-analysis to compare the cumulative opioid consumption, pain intensity and incidence of complications after surgery between the pregabalin group and the placebo group to provide recommendations for surgeons and anesthesiologists.

## Materials and methods

### Inclusion and exclusion criteria

This meta-analysis was performed following the PRISMA guidelines (www.prisma-statement.org) and was registered on 10 November 2021 with INPLASY (registration number: INPLASY2021110031) (Details were displayed in Additional file 1: Appendix 1). The studies were selected based on the PICO criteria. Studies comparing the outcomes of cumulative opioid consumption, pain intensity or complication incidence following preoperative administration of oral pregabalin and placebo in patients undergoing lower limb orthopedic surgery were included in the current analysis.

### Search strategy

The PubMed, EMBASE, and Cochrane Library databases were systematically searched (search trials are displayed in Additional file 2: Appendix 2). No restrictions on the publication status or language were applied.

Studies were assessed by two of the authors independently, and data were extracted with a standard data extraction form. The risk of bias was assessed for the included studies based on the Cochrane risk-of-bias criteria. Any disagreement was resolved through discussion.

### Statistical analysis

The primary outcomes in this analysis were defined as the cumulative opioid consumption, static pain intensity and dynamic pain intensity. For opioid consumption, the reported data were converted to the oral morphine equivalent dose. For pain intensity, the data of VAS (0, no pain; 10, worst imaginable pain) were pooled. The secondary outcomes included the incidence of complications, such as nausea, vomiting, dizziness and sedation. Mean differences (MD) with a 95% CI were calculated using the inverse variance method for continuous variables, and risk ratios (RR) with a 95% CI were calculated using the Mantel–Haenszel analysis method for dichotomous variables. Heterogeneity was assessed using the Chi^2^ and *I*^2^ tests, and an *I*^2^ of > 50% was identified as substantial heterogeneity. Sensitivity analysis was performed for variables presenting with substantial heterogeneity by sequentially excluding individual studies. Subgroup analysis was conducted by stratifying studies according to dosing regimen (receiving pregabalin preoperatively only vs receiving pregabalin both pre- and postoperatively) and pregabalin dose (> 150 mg/day vs ≤ 150 mg/day). We used the Chi^2^ test to test for subgroup interactions. Analysis was undertaken using RevMan 5.4 (The Nordic Cochrane Center, The Cochrane Collaboration, Copenhagen, Denmark) with a significance threshold of *P* < 0.05.

## Results

### Study retrieved and characteristics

**Fig. 1:**
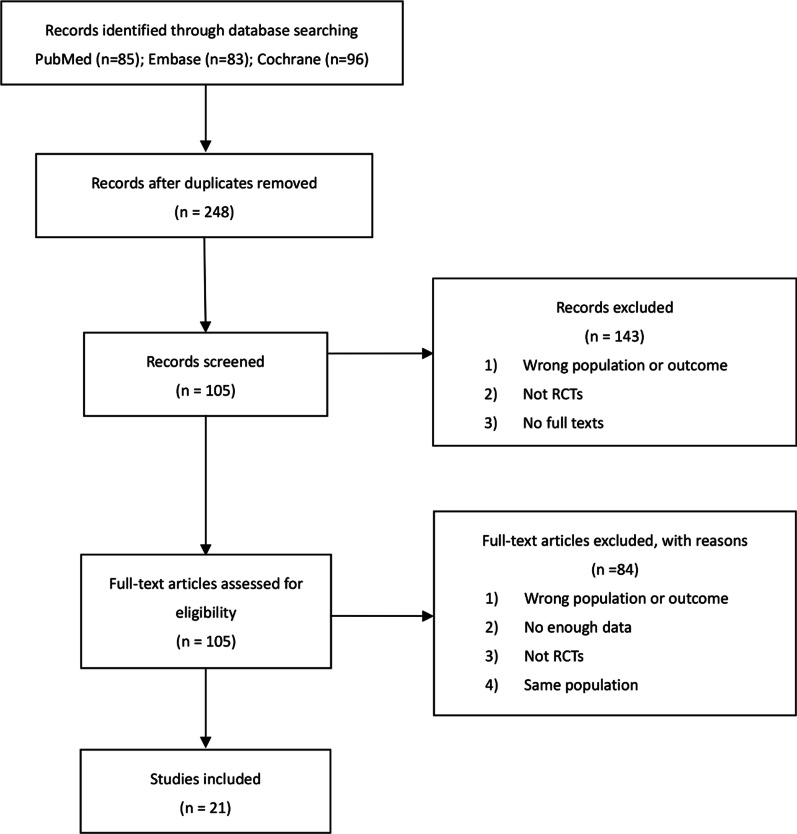
Flow diagram

Twenty-one randomized controlled trials from 11 countries with a total sample size of 1520 (453 for the pregabalin group, 1067 for the placebo group) were included in the current analysis (Fig. 1) [12–31]. Characteristics including the author’s name, year, origin, anesthesia, and dosing regimen were extracted and are displayed in Table 1. The studies by Nimmaanrat et al. and Yik et al. were not included in the quantitative analysis because of the inconsistent results they reported [26, 31]. The studies by Martinez et al. and Niruthisard et al. were treated as two comparisons due to the combined use of other drugs [14, 27].

**Table 1:** Characteristics of the included studies

Studies	Origin	Anesthesia	Sample size		Medication plan	
			Control	Pregabalin	Preoperative	Postoperative
Kheirabadi 2020	Iran	Spinal anesthesia	24	26	75-mg 1 h preop	Nil
Damirchi 2019	Iran	NA	25	25	150-mg 2 h preop	Nil
Sebastian 2016	India	Spinal anesthesia	45	45	150-mg 1 h preop	Nil
Khetarpal 2016	India	Spinal anesthesia	30	30	300-mg 1.5 h preop	Nil
Akhavanakbari 2013	Iran	Spinal anesthesia	30	30	150-mg 2 h preop	Nil
Yadeau 2012	USA	Spinal anesthesia and peripheral nerve blockade	28	28	100-mg 1 h preop	50-mg pregabalin Bid for 3 days postop
Buvanendran 2010	USA	Spinal anesthesia	110	106	300-mg 1–2 h preop	150-mg Bid for days 1–10, 75-mg Bid for days 11 and 12, 50-mg Bid for days 13 and 14 postop
Jain 2012	India	Spinal anesthesia	20	20	75-mg 2 h preop	75-mg Bid for 2 days postop
Singla 2015	USA	Spinal or epidural anesthesia	104	Arm 1 (n = 103)	75-mg 12 h and 2 h preop	75-mg Bid for 6 weeks postop
				Arm 2 (n = 100)	150-mg 12 h and 2 h preop	150-mg Bid for 6 weeks postop
Yadeau 2015	USA	Spinal and epidural anesthesia and peripheral nerve blockade	28	Arm 1 (n = 26)	100-mg 30 min preop	50-mg Bid for days 1 to 14, 50-mg Qd for days 15 and 16 postop
				Arm 2 (n = 29)	200-mg 30 min preop	100-mg Bid for days 1 to 14, 100-mg Qd for days 15 and 16 postop
				Arm 3 (n = 28)	300-mg 30 min preop	150-mg Bid for days 1 to 14, 150-mg Qd for days 15 and 16 postop
Mathiesen 2008	Denmark	Spinal anesthesia	38	40	300-mg 1 h before anesthesia	Nil
Clarke 2015	Canada	Spinal anesthesia	79	83	150-mg 2 h preop	75-mg Bid throughout hospital stay and for 7 days after discharge
Martinez 2014	France	General anesthesia	38	Comparison 1 (n = 35)	150-mg 2 h preop	Nil
			34	Comparison 2 (n = 35)	150-mg 2 h preop, plus intravenous ketamine	Nil
Yik 2019	Singapore	General anesthesia	42	45	75-mg preop	75-mg Qd for 2 days postop
Rahat 2018	Iran	Spinal anesthesia	60	60	150-mg 1 h before anesthesia	Nil
Omara 2019	Egypt	Spinal anesthesia	30	30	150-mg 1 h preop	Nil
Nimmaanrat 2012	Thailand	Spinal anesthesia	29	27	75-mg 1 h before anesthesia	75-mg 12 h after the first dose
Lee 2015	Korea	General anesthesia	20	21	150-mg 1 h preop	Nil
Kavak 2020	Turkey	Spinal anesthesia	18	16	150-mg 1 h before anesthesia	Nil
Niruthisard 2013	Thailand	Spinal anesthesia	27	Comparison 1 (n = 25)	150-mg 2 h preop	Nil
			24	Comparison 2 (n = 24)	150-mg 2 h preop, plus celecoxib 400-mg	Nil
Buvanendran 2012	USA	Spinal anesthesia	14	Arm 1 (n = 14)	150-mg 1 h preop	Nil
				Arm 2 (n = 16)	150-mg 24 h, 12 h and 1 h preop	Nil

### Study quality assessment

**Fig. 2:**
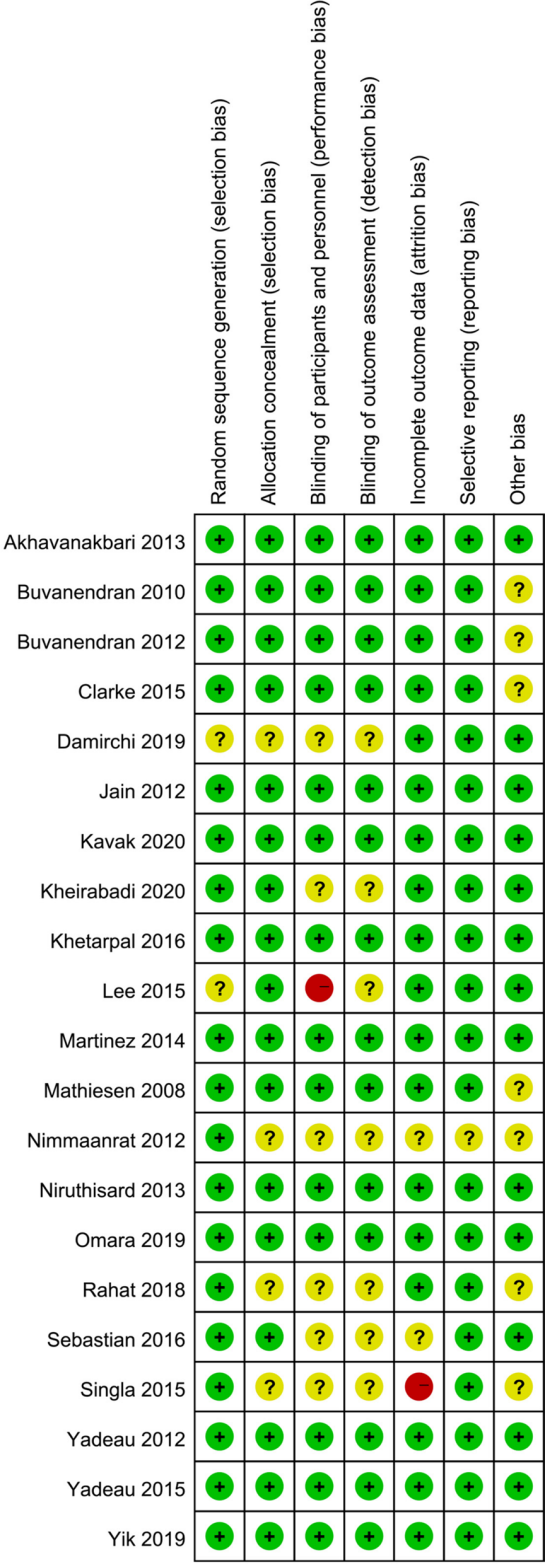
Risk of bias summary

In the current analysis, we defined the trials as high quality when selection bias was graded as low risk, with the others as low or unclear. When selection bias was assessed as high risk, trials were graded as low quality. Trials were defined as moderate quality if they did not meet the above criteria. Sixteen trials were graded as high quality [12–14, 16–18, 20–23, 25, 27, 28, 30–32], and five trials were graded as moderate quality [15, 19, 24, 26, 29]. The details of the quality assessment are displayed in Fig. 2.

### Cumulative opioid consumption at 24 h and 48 h postoperatively

**Fig. 3:**
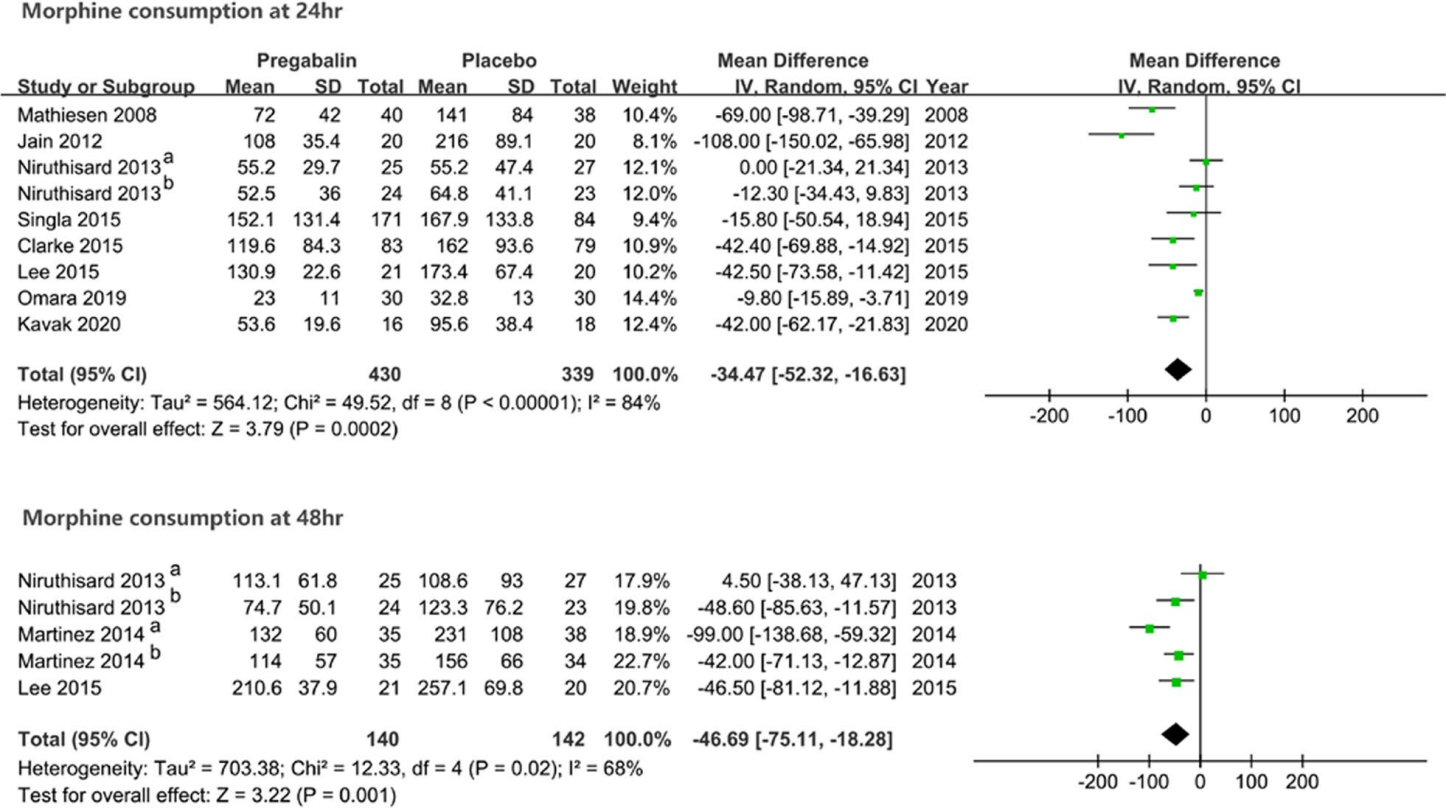
The forest plot shows the comparison results in cumulative opioid consumption within 24 and 48 h after surgery between groups

The reported opioid consumption was uniformly converted to the oral morphine equivalent dose. Patients who received pregabalin preoperatively had significantly lower opioid consumption than patients receiving placebo at 24 h after surgery (MD − 34.47, 95% CI [− 52.32, − 16.63], *P* = 0.0002), and substantial heterogeneity was observed (I^2^ = 84%). For the results at 48 h after surgery, a similarly lower morphine equivalent dose was detected in patients receiving pregabalin than in those receiving placebo (MD − 46.69, 95% CI [− 75.11, − 18.28], *P* = 0.001). High heterogeneity was also seen (*I*^2^ = 68%) (Fig. 3). Sensitivity analyses were performed for the results, and no individual studies detected a significant influence on the pooled results of morphine consumption within 24 and 48 h (details are displayed in Additional file 3: Appendix 3).

### Postoperative pain intensity

**Fig. 4:**
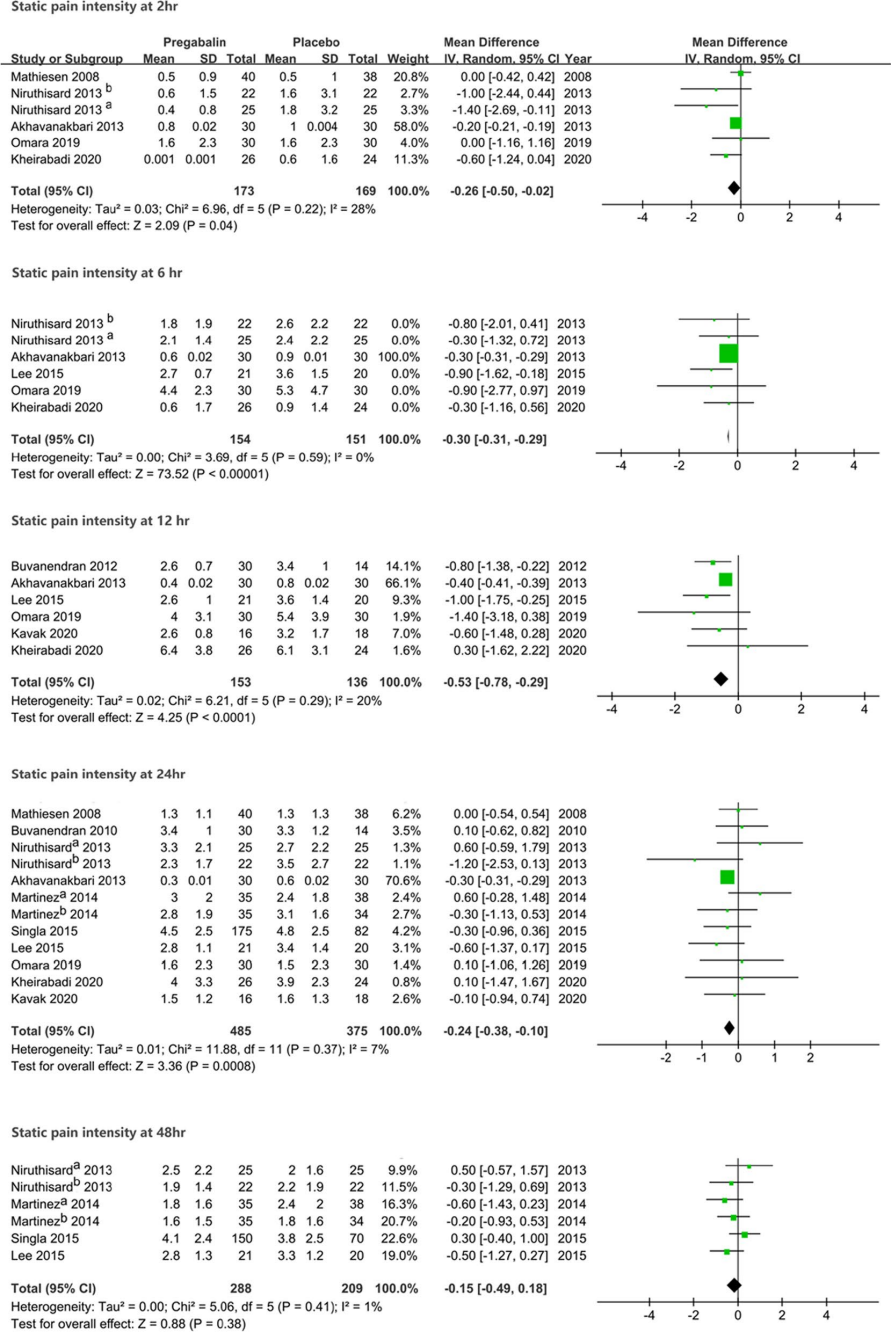
The forest plot shows the comparison results in pain intensity at rest at 2, 6, 12, 24 and 48 h after surgery between groups

Significantly lower static pain intensity favoring patients who received pregabalin preoperatively was noted at 2 h (MD − 0.26, 95% CI [− 0.50, − 0.02], *P* = 0.04), 6 h (MD − 0.30, 95% CI [− 0.31, − 0.29], *P* < 0.00001), 12 h (MD − 0.53, 95% CI [− 0.78, − 0.29], *P* < 0.0001), and 24 h (MD − 0.24, 95% CI [− 0.38, − 0.10], *P* = 0.0008). However, no difference in static pain intensity was observed at 48 h between groups (MD − 0.15, 95% CI [− 0.49, 0.18], *P* = 0.38), with no substantial heterogeneity seen for static pain intensity (Fig. 4).

**Fig. 5:**
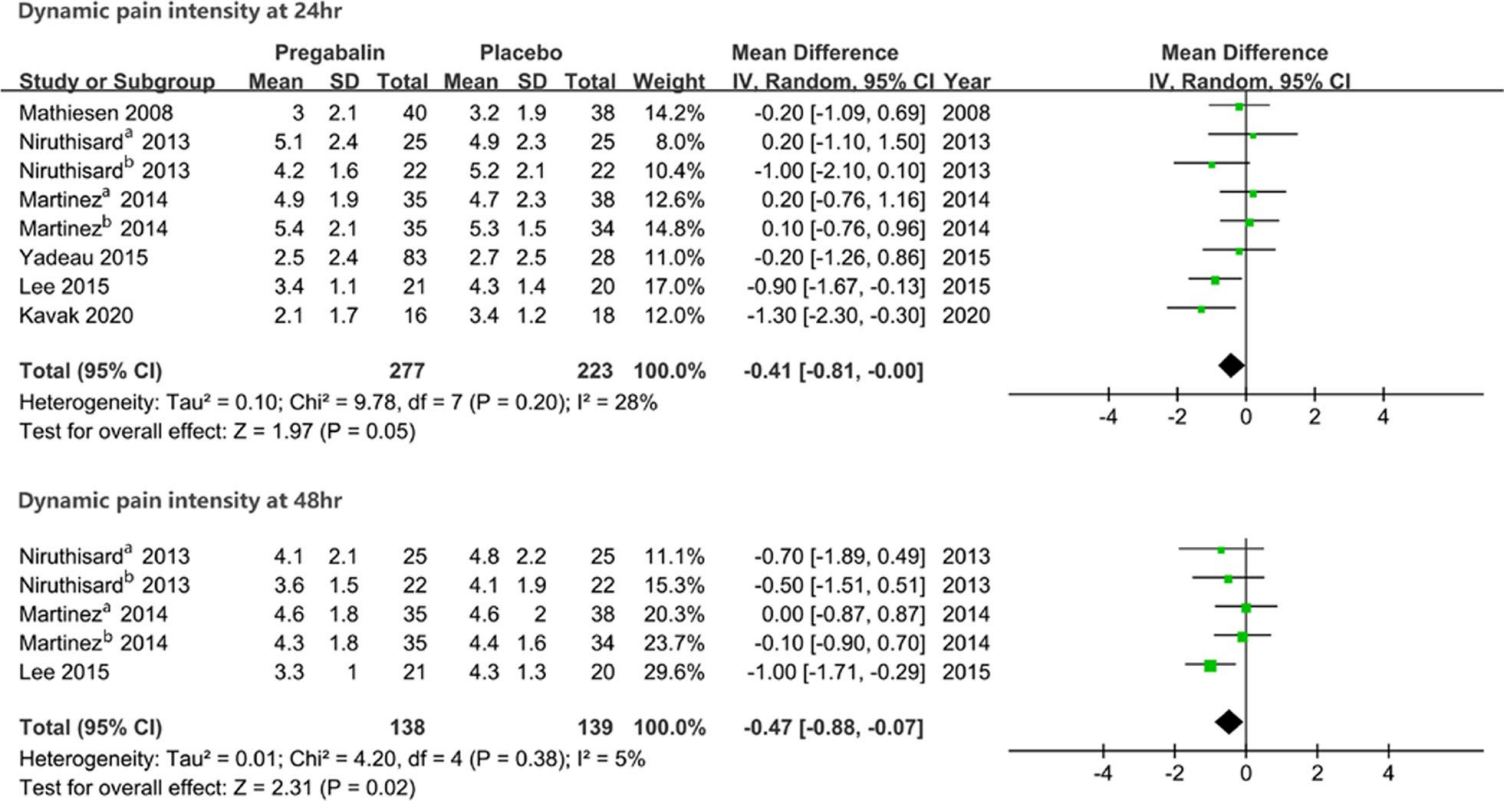
The forest plot shows the comparison results in pain intensity on movement at 24 and 48 h after surgery between groups

Patients who received preoperative pregabalin had significantly lower dynamic pain intensity than patients receiving placebo at 48 h (MD − 0.47, 95% CI [− 0.88, − 0.07], *P* = 0.02). However, no difference in dynamic pain intensity at 24 h was seen between groups (MD − 0.41, 95% CI [− 0.81, 0.00], *P* = 0.05), with no heterogeneity seen for dynamic pain intensity (Fig. 5).

### Incidence of complications

**Fig. 6:**
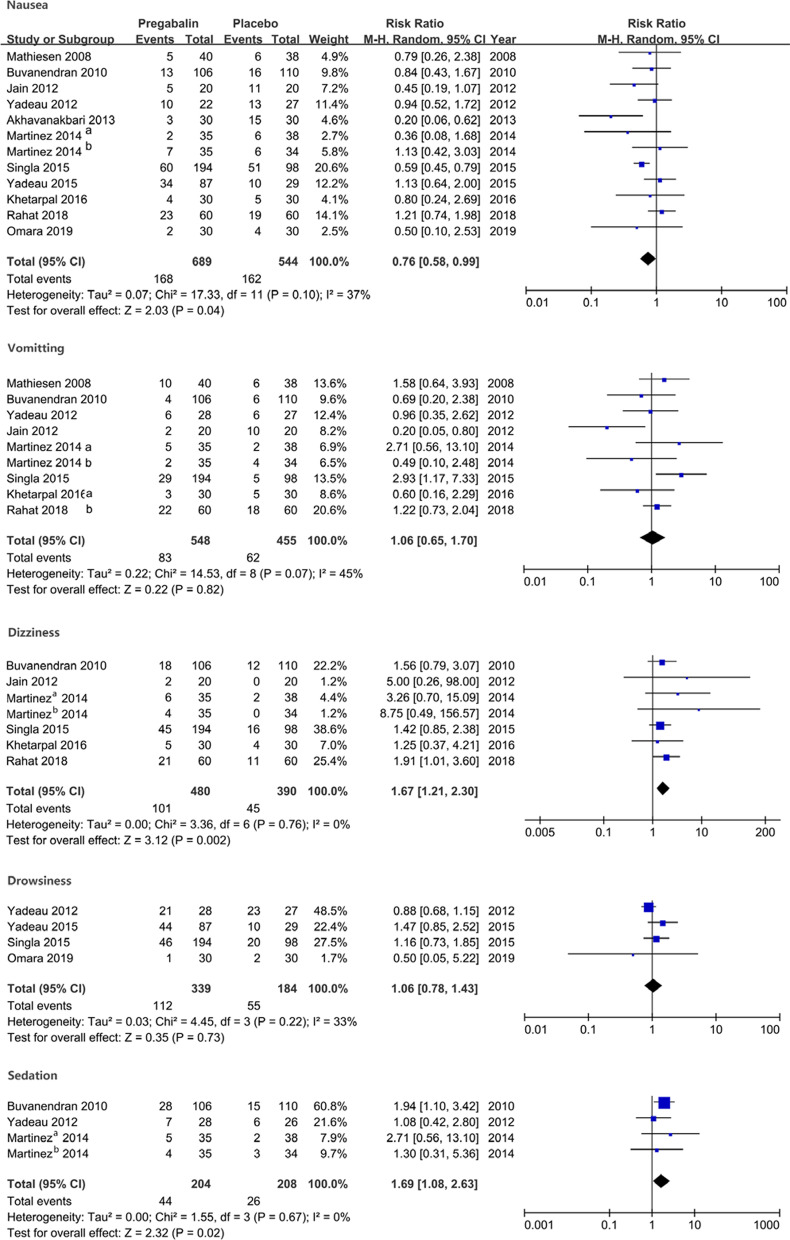
The forest plot shows the comparison results for the incidence of complications between groups

Preoperative use of pregabalin lowered the incidence of nausea after surgery (RR 0.76, 95% CI [0.58, 0.99], *P* = 0.04). An increased postoperative incidence of dizziness and sedation was observed in patients treated with pregabalin (RR 1.67, 95% CI [1.21, 2.30], *P* = 0.002) for the incidence of dizziness; RR 1.69, 95% CI [1.08, 2.63], *P* = 0.03 for the incidence of sedation). No difference in the incidence of vomiting and drowsiness was seen in patients receiving pregabalin (RR 1.06, 95% CI [0.65, 1.70], *P* = 0.82 in the incidence of vomiting; RR 1.04, 95% CI [0.64, 1.69], *P* = 0.87 in the incidence of drowsiness). No substantial heterogeneity was seen for the outcomes of complications (Fig. 6).

Two trials included in this study reported the outcomes of chronic neuropathic pain assessed by the Leeds Assessment of Neuropathic Symptoms and Signs pain scale (LANSS) [13, 17]. Quantitative analysis of chronic pain was not performed because of the paucity of data.

### Subgroup analyses

To compare the effectiveness of pregabalin in patients taking various dosing regimens, subgroup analyses were performed. A minimum of two comparisons per subgroup were available in the current analysis. There was no statistically significant difference in the results of cumulative opioid consumption within 24 h (test for subgroup differences, *P* = 0.30) in the subgroups of patients receiving pregabalin only before surgery or in patients receiving pregabalin both before and after surgery. Similarly, no significant difference between subgroups was shown in the results of static pain intensity at 24 h and incidence of complications. Details are displayed in Table 2.

**Table 2:** Subgroup analysis in studies stratified by dosing regimen

Subgroup	No. of comparisons	Sample size	Mean difference or risk ratio (95% CI)	Test for subgroup differences
		(Pregabalin/Placebo)		*(P* value)
Static pain intensity at 24 h				
Dosing regimen A	10	280/279	− 0.21 [− 0.41, − 0.00]	0.75
Dosing regimen B	2	205/96	− 0.12 [− -0.61, 0.37]	
Morphine consumption within 24 h				
Dosing regimen A	6	156/156	− 26.77 [− 44.90, − 8.65]	0.3
Dosing regimen B	3	274/183	− 53.72 [− 100.88, − 6.57]	
Nausea				
Dosing regimen A	7	260/260	0.70 [0.41, 1.19]	0.84
Dosing regimen B	5	429/284	0.75 [0.55, 1.02]	
Vomiting				
Dosing regimen A	5	200/200	1.20 [0.81, 1.78]	0.56
Dosing regimen B	4	348/255	0.86 [0.30, 2.48]	
Dizziness				
Dosing regimen A	4	160/162	1.97 [1.17, 3.32]	0.42
Dosing regimen B	3	320/228	1.50 [1.00, 2.26]	
Sedation				
Dosing regimen A	2	70/72	1.81 [0.63, 5.18]	0.88
Dosing regimen B	2	134/136	1.65 [0.99, 2.75]	

Dosing regimen A, receiving pregabalin preoperatively only; Dosing regimen B, receiving pregabalin both pre- and postoperatively

To further explore the effect of pregabalin dose in patients undergoing lower limb orthopedic surgery, post hoc subgroup analyses were conducted in studies stratified by pregabalin dose (> 150 mg/day vs ≤ 150 mg/day). A minimum of two arms per subgroup was available in the current analysis. There was no statistically significant difference in opioid consumption within 24 h (test for subgroup differences, *P* = 0.62) between subgroups. Comparable results between subgroups were also detected in static pain intensity at 24 h (test for subgroup differences, *P* = 0.30), dynamic pain intensity at 24 h (test for subgroup differences, *P* = 0.72) and incidence of complications. Details are displayed in Table 3.

**Table 3:** Subgroup analysis in studies stratified by pregabalin dose

Subgroup	No. of arms	Sample size	Mean difference or risk ratio (95% CI)	Test for subgroup differences
		(pregabalin/placebo)		*(P* value)
Static pain intensity at 24 h				
≥ 150 mg/day	2	70/52	0.04 [− 0.40, 0.47]	0.3
< 150 mg/day	9	240/241	− 0.22 [− 0.46, 0.01]	
Dynamic pain intensity at 24 h				
≥ 150 mg/day	3	97/94	− 0.26 [− 0.89, 0.37]	0.72
< 150 mg/day	7	180/185	− 0.41 [− 0.91, 0.08]	
Morphine consumption within 24 h				
≥ 150 mg/day	2	126/122	− 44.06 [− 96.28, 8.16]	0.62
< 150 mg/day	8	304/301	− 30.12 [− 47.65, − 12.60]	
Nausea				
≥ 150 mg/day	6	330/334	0.77 [0.60, 0.99]	0.77
< 150 mg/day	9	359/366	0.72 [0.50, 1.04]	
Vomiting				
≥ 150 mg/day	4	272/276	0.77 [0.49, 1.21]	0.99
< 150 mg/day	6	276/277	0.78 [0.44, 1.37]	
Drowsiness				
≥ 150 mg/day	3	154/156	1.43 [1.02, 1.98]	0.05
< 150 mg/day	4	339/340	0.95 [0.76, 1.19]	
Dizziness				
≥ 150 mg/day	3	232/238	1.54 [1.02, 2.31]	0.73
< 150 mg/day	5	248/250	1.70 [1.13, 2.57]	

## Discussion

The main findings of the current analysis were that the use of pregabalin preoperatively in patients undergoing lower limb orthopedic surgery appeared to be associated with lower morphine consumption at 24 h and 48 h. Similarly, decreased static pain intensity within 24 h and dynamic pain intensity at 48 h were detected in patients taking pregabalin. Compared with placebo, the use of pregabalin was associated with a reduction in the incidence of nausea but an increase in dizziness. No evidence was found with subgroup analyses to support the continued use of pregabalin postoperatively or using more than 150 mg of pregabalin per day.

To our knowledge, this was the first meta-analysis focused on the efficacy of pregabalin in perioperative pain management in patients undergoing lower limb orthopedic surgery. The primary outcomes in the current analysis were pain intensity and cumulative opioid consumption. A small reduction in the pain intensity at rest, as described in existing research [33], was detected in patients receiving pregabalin at all time points within the first day after surgery. Similar outcomes of pain intensity on movement at 48 h were also found. In addition, lower heterogeneity was noted among the studies. All the aforementioned outcomes confirm the analgesic effectiveness of pregabalin in patients undergoing lower limb orthopedic surgery, which has been known to induce hyperalgesia [34]. The test for subgroup differences was not significant, suggesting that altered pregabalin dose or dosing regimen does not affect the analgesic effectiveness of pregabalin.

Significantly lower morphine consumption within 24 h and 48 h was observed in patients receiving pregabalin, indicating that preoperative pregabalin could effectively reduce opioid consumption postoperatively. A similar result was also detected in a study by Lee et al. [24], who reported that pregabalin led to a reduction in fentanyl consumption during the first or the second days after surgery. Considering the reduction in pain intensity, the opioid-sparing effect of pregabalin can be regarded as a manifestation of analgesic effectiveness. Substantial heterogeneity was observed in the results, and sensitivity analyses were performed by subsequently excluding individual studies. However, no individual studies detected a significant influence on these pooled results, suggesting that the results were stable. Subgroup analyses were conducted on morphine consumption within 24 h by stratifying studies according to the pregabalin dose and dosing regimen. The heterogeneity persisted in each subgroup, suggesting that variations in pregabalin dose and dosing regimen could not explain the heterogeneity in opioid consumption. Although we converted the reported data to the oral morphine equivalent dose uniformly, the variations in the types of opioids and their routes of administration may be the possible source of heterogeneity in opioid consumption. Other possible explanations may be the various surgical sites, incision lengths, surgical experiences, perioperative analgesic regimens, and patient factors. However, it was impossible to perform subgroup analyses with regard to the aforementioned items because of the paucity of data. Additionally, the test for subgroup differences (stratified by pregabalin dose and dosing regimen) was not significant, suggesting that an increased pregabalin dose or long-term dosing regimen seemed to be ineffective in further reducing the consumption of opioids.

Recently, perioperative pain management has sought to reduce the incidence of opioid-related adverse effects [33]. In the current analysis, a lower incidence of nausea was observed in patients receiving pregabalin, indicating that pregabalin can reduce the incidence of opioid-related adverse effects through its opioid-sparing effect. The incidence of dizziness and sedation, however, was significantly higher in the pregabalin group, which is considered to be an adverse effect associated with pregabalin [35]. Hence, we should use pregabalin with caution. Moreover, the test for subgroup differences regarding the results of dizziness stratified by pregabalin dose was not significant, indicating that pregabalin had no dose effect on the incidence of dizziness. These results are contrary to the outcomes of previous research, which reported that the adverse effects of pregabalin are dose-dependent [36]. The difference in results may be due to the variations in the types of surgery. Moreover, considering the indirect comparisons that existed in our current analysis, we cannot exclude a possible dose effect of pregabalin on adverse effects. Hence, considering that there is no detectable difference in the results of pregabalin’s analgesic effect and opioid-sparing effect between subgroups stratified by pregabalin dose, we recommend that the use of pregabalin does not exceed 150 mg per day.

Only two trials that reported the incidence of chronic neuropathic pain were identified in this analysis. Formal meta-analysis was not performed because of the paucity of data. Buvanendran et al. [17] reported a lower incidence of chronic neuropathic pain in patients receiving pregabalin. However, no significant reduction in the incidence of chronic neuropathic pain was noted in the study by Yadeau et al. [13]. At present, no clear evidence has been proposed with regard to the beneficial effects of pregabalin on the prevention of chronic neuropathic pain. More research that focuses on chronic neuropathic pain is needed to solve this problem.

Several limitations were detected in the current analysis. First, substantial heterogeneity among studies was observed regarding the results of morphine consumption, and further studies are needed to enhance the strength of the evidence or find the source of heterogeneity. Second, comparisons of the outcomes stratified by pregabalin dose or dosing regimen were indirect in the current analysis, and studies that compared different doses or dosing regimens directly would have to further evaluate the optimal dosage and dosing regimen of pregabalin. Third, the data presented in some studies were not suitable for pooling for meta-analyses. Finally, although the results were statistically significant, whether it is clinically significant requires further investigation, randomized controlled trials with a larger sample size are needed in future work.

In summary, this meta-analysis supports the use of pregabalin preoperatively in patients undergoing lower limb orthopedic surgery with regard to opioid-sparing and analgesic effects. However, it is wary of the resulting increase in the incidence of dizziness and sedation. At present, there is no evidence to recommend the continued use of pregabalin postoperatively or the use of more than 150 mg of pregabalin per day.

## Supplementary Information

**Additional file 1**. The details of the registration information.

**Additional file 2**. The details of the search trial.

**Additional file 3**. The results of the sensitivity analyses,

## Data Availability

As a meta-analysis, there are no patient data sets.
