# Supplementary material for: The preemptive effects of oral pregabalin on perioperative pain management in lower limb orthopedic surgery: a systematic review and meta-analysis
Source: J Orthop Surg Res. 2022 Apr 13;17:237. doi: 10.1186/s13018-022-03101-9 (PMC9006545; doi:10.1186/s13018-022-03101-9)
Supplement: Supplementary file 1 — Additional file 1. The details of the registration information. [file 13018_2022_3101_MOESM1_ESM.pdf]

INPLASY PROTOCOL

To cite: Chen et al. The preemptive effects of oral pregabalin on perioperative pain management in lower limb orthopedic surgery: a systematic review and meta-analysis. Inplasy protocol 2021110031. doi: 10.37766/inplasy2021.11.0031

Received: 09 November 2021

Published: 09 November 2021

Corresponding author:
Zhou Xiang

xiangzhou13@hotmail.com

Author Affiliation:
Sichuan University.

Support: **National Natural Science Found.**

Review Stage at time of this submission: **Data analysis.**

Conflicts of interest:
None declared.

The preemptive effects of oral pregabalin on perioperative pain management in lower limb orthopedic surgery: a systematic review and meta-analysis

Chen, Z[1]; Xiang, Z[2]; Chen, J[3]; Luo, R[4].

Review question / Objective: To review the literatures systematically and make a comprehensive understanding of the preemptive effects of oral pregabalin on perioperative pain management in lower limb orthopedic surgery.
Condition being studied: Pregabalin has been used in lower limb orthopedic surgery for more than ten years, several previous randomized trials have dedicated to exploring its effect on perioperative pain management. While there is no official consensus as to whether pregabalin is effective in perioperative pain management for patients undergoing lower limb orthopedic surgery. A meta-analysis is warranted.

INPLASY registration number: This protocol was registered with the International Platform of Registered Systematic Review and Meta-Analysis Protocols (INPLASY) on 09 November 2021 and was last updated on 09 November 2021 (registration number INPLASY2021110031).

INTRODUCTION

Review question / Objective: To review the literatures systematically and make a comprehensive understanding of the preemptive effects of oral pregabalin on perioperative pain management in lower limb orthopedic surgery.

Condition being studied: Pregabalin has been used in lower limb orthopedic surgery for more than ten years, several previous randomized trials have dedicated to

Chen et al. Inplasy protocol 2021110031. doi:10.37766/inplasy2021.11.0031 Downloaded from https://inplasy.com/inplasy-2021-11-0031/

exploring its effect on perioperative pain management. While there is no official consensus as to whether pregabalin is effective in perioperative pain management for patients undergoing lower limb orthopedic surgery. A meta-analysis is warranted.

METHODS

Participant or population: Patients undergoing lower limb orthopedic surgery.

Intervention: Pregabalin vs placebo.

Comparator: Pain intensity, opioids consumption, incidence of complications.

Study designs to be included: Randomized controlled trials.

Eligibility criteria: Studies compared the outcomes of cumulative opioid consumption, pain intensity or complications incidence following preoperative administration of oral pregabalin and placebo in patients undergoing lower limb orthopedic surgery were included in the current analysis.

Information sources: Electronic databases (PubMed, EMBASE, and Cochrane library).

Main outcome(s): Cumulative oral morphine consumption within 24 hours and 48 hours; Pain intensity at rest at 2, 6, 12, 24, and 48 hours; Pain intensity on movement at 24 and 48 hours; Incidence of complications (nausea, vomiting, drowsiness, dizziness, sedation).

Quality assessment / Risk of bias analysis: The risk of bias was assessed for included studies based on the Cochrane risk-of-bias criteria.

Strategy of data synthesis: Mean differences (MD) with a 95% CI were calculated using the inverse variance method for continuous variables, and risk ratio (RR) with a 95% CI were calculated using the Mantel-Haenszel analysis method for dichotomous variables.

Subgroup analysis: Subgroup analyses were performed by stratifying studies according to the pregabalin dose and dosing regimen.

Sensitivity analysis: Sensitivity analyses for variables presenting with substantial heterogeneity were conducted by sequentially excluding individual studies.

Country(ies) involved: China.

Keywords: Pregabalin; Perioperative pain management; Lower limb orthopedic surgery; Meta-analysis.

Contributions of each author:
Author 1 - Zhao Chen.
Author 2 - Zhou Xiang.
Author 3 - Jialei Chen.
Author 4 - Rong Luo.

Chen et al. Inplasy protocol 2021110031. doi:10.37766/inplasy2021.11.0031 Downloaded from https://inplasy.com/inplasy-2021-11-0031/
