# Supplementary material for: The preemptive effects of oral pregabalin on perioperative pain management in lower limb orthopedic surgery: a systematic review and meta-analysis
Source: J Orthop Surg Res. 2022 Apr 13;17:237. doi: 10.1186/s13018-022-03101-9 (PMC9006545; doi:10.1186/s13018-022-03101-9)
Supplement: Supplementary file 2 — Additional file 2. The details of the search trial. [file 13018_2022_3101_MOESM2_ESM.docx]

| 1. "Pregabalin"[Mesh] |
| --- |
| 1. " Gamma-Aminobutyric Acid "[Mesh] |
| 1. Pregabalin [Title/Abstract] |
| 1. gamma-Aminobutyric Acid [Title/Abstract] |
| 1. OR/1-4 |
| 1. "Lower Extremity"[Mesh] 2. "Arthroplasty, Replacement"[Mesh] |
| 1. Lower limb orthopedic surgery [Title/Abstract] |
| 1. Lower extremity orthopedic surgery [Title/Abstract] 2. Femur surgery [Title/Abstract] 3. Tibia surgery [Title/Abstract] 4. Fibula surgery [Title/Abstract] 5. Ankle surgery [Title/Abstract] 6. Foot surgery [Title/Abstract] 7. Arthroplasty"[Title/Abstract] 8. Replacement"[Title/Abstract] |
| 1. OR/6-16 |
| 1. Randomized controlled trial [Publication Type] 2. Controlled clinical trial [Publication Type] 3. Comparative study [Publication Type] 4. OR/18-20 5. 5 AND 17 AND 21 |
