# Supplementary material for: The preemptive effects of oral pregabalin on perioperative pain management in lower limb orthopedic surgery: a systematic review and meta-analysis
Source: J Orthop Surg Res. 2022 Apr 13;17:237. doi: 10.1186/s13018-022-03101-9 (PMC9006545; doi:10.1186/s13018-022-03101-9)
Supplement: Supplementary file 3 — Additional file 3. The results of the sensitivity analyses, [file 13018_2022_3101_MOESM3_ESM.docx]

| Table A. Sensitivity analysis for results of opioids consumption within 24 hours | | |
| --- | --- | --- |
| Study | Mean difference (95% CI) | I²-statistics (%) |
| Omitting Mathiesen 2008 | -29.88 [-47.19, -12.57] | 81 |
| Omitting Jain 2012 | -27.36 [-42.78, -11.94] | 78 |
| Omitting Niruthisard^a^ 2013 | -39.59 [-59.76, -19.42] | 85 |
| Omitting Niruthisard^b^ 2013 | -37.97 [-58.47, -17.47] | 86 |
| Omitting Singla 2015 | -36.68 [-56.12, -17.24] | 86 |
| Omitting Clarke 2015 | -33.63 [-52.88, -14.37] | 85 |
| Omitting Lee 2015 | -33.67 [-52.81, -14.53] | 85 |
| Omitting Omara 2019 | -38.98 [-59.76, -18.21] | 79 |
| Omitting Kavak 2020 | -33.60 [-53.17, -14.03] | 84 |

| Table B. Sensitivity analysis for results of opioids consumption within 48 hours | | |
| --- | --- | --- |
| Study | Mean difference (95% CI) | I²-statistics (%) |
| Omitting Lee 2015 | -46.65 [-83.84, -9.45] | 76 |
| Omitting Martinez^a^ 2014 | -35.84 [-57.26, -14.41] | 32 |
| Omitting Martinez^b^ 2014 | -47.89 [-86.51, -9.27] | 75 |
| Omitting Niruthisard^a^ 2013 | -57.06 [-81.34, -32.77] | 49 |
| Omitting Niruthisard^b^ 2013 | -46.13 [-82.67, -9.59] | 76 |
